# Biophysical aspects of mechanotransduction in cells and their physiological/biological implications in vocal fold vibration: a narrative review

**DOI:** 10.3389/fcell.2025.1501341

**Published:** 2025-01-27

**Authors:** Junseo Cha, Susan L. Thibeault

**Affiliations:** Department of Surgery, Division of Otolaryngology-Head and Neck Surgery, University of Wisconsin–Madison, Madison, WI, United States

**Keywords:** mechanotransduction, mechanosensitive channels, vocal fold mimetic bioreactor, vocal fold vibrations, mechanical stimulation

## Abstract

Mechanotransduction is a crucial property in all organisms, modulating cellular behaviors in response to external mechanical stimuli. Given the high mobility of vocal folds, it is hypothesized that mechanotransduction significantly contributes to their tissue homeostasis. Recent studies have identified mechanosensitive proteins in vocal fold epithelia, supporting this hypothesis. Voice therapy, which, involves the mobilization of vocal folds, aims to rehabilitate vocal function and restore homeostasis. However, establishing a direct causal link between specific mechanical stimuli and therapeutic benefits is challenging due to the variability in voice therapy techniques. This challenge is further compounded when investigating biological benefits in humans. Vocal fold tissue cannot be biopsied without significant impairment of the vibratory characteristics of the vocal folds. Conversely, studies using vocal fold mimetic bioreactors have demonstrated that mechanical stimulation of vocal fold fibroblasts can lead to highly heterogeneous responses, depending on the nature and parameters of the induced vibration. These responses can either aid or impede vocal fold vibration at the physiological level. Future research is needed to determine the specific mechanical parameters that are biologically beneficial for vocal fold function.

## Introduction

It is well established that phonotrauma can result in vocal fold inflammation, hemorrhage, and development of mass lesions (Kleinsasser, 1982; Abitbol, 1988; Courey et al., 1996). When vocal folds are in prolonged intensive motion, mechanical stimuli bring about biological changes such as the destruction of the epithelial layer (Kojima et al., 2014; Kimball et al., 2021), and secretion of pro-inflammatory markers (Verdolini et al., 2003; Li et al., 2011; Verdolini-Abbott et al., 2012) which represent the initial stage of wound healing. While some limited evidence exists that mobilizing the vocal folds with voice therapy may be more beneficial than complete rest (Verdolini-Abbott et al., 2012), recent reviews in this area show that voice therapy protocols vary significantly in how mechanical stimulations are imposed on the vocal folds along with treatment duration (Desjardins et al., 2017; Alegria et al., 2020; Barsties et al., 2020). Furthermore, treatment efficacy from voice therapy becomes more ambiguous due to different outcome measures. So far, very little is understood about vocal fold mobilization and its biological impact on the micro and macrostructure of the vocal folds. Understanding the biological effects of induced mechanical stimuli on vocal fold tissues is crucial and has the potential to inform the underlying mechanism behind voice therapies and wound-healing processes.

When mechanical stimuli are induced, cells within the vocal folds shift their behaviors through mechanotransduction, leading to biological and physiological changes at the macroscopic level. Mechanotransduction refers to the process by which biochemical intracellular changes occur after external mechanical stimuli are induced to cells. Mechanical stimuli can be any sort of external force, including stretching tension, shear force, compressive force, and hydrostatic or osmotic pressures. It is believed that the ability to sense mechanical stimuli originates very early in organisms, as it is highly preserved in all Bacteria, Archaea, and Eukarya domains (Martinac and Kloda, 2003). Understandably, mechanotransduction has proven to be a critical property in maintaining homeostasis, such as in the cardiovascular system or in bone structure formation, where the ability to sense shear stresses resulting from fluid flow and movement is critical for regulating vascular pressure and osteogenesis (Lammerding et al., 2004; Haga et al., 2007; Wang et al., 2009). It has been recognized that mechanotransduction is achieved by activating mechanosensitive (MS) channels located on the surface of cells. These gated MS channels open upon activation, leading to an influx of ions such as K^+^, Ca^2+^, and Na^+^. The influx of these ions then acts as modulators in downstream intracellular changes that ultimately influence cell migration, apoptosis, differentiation, proliferation, and gene expressions (Mayr et al., 2000; Grossi et al., 2007; Zhu et al., 2023). Some MS channels are known to be non-selective cationic channels, such as the PIEZO and most TRP superfamily channels (Owsianik et al., 2006; Coste et al., 2010). In contrast, other channels, such as Shaker (Kv 1.1), TREK1, TRAAK, and NAV 1.5, are known to be selective channels to a single type of ions (Maingret et al., 1999; Beyder et al., 2010; Brohawn et al., 2012; Hao et al., 2013). To be considered as an MS channel, Arnadóttir and Chalfie (2010) proposed four criteria: 1) channel must be expressed temporally and spatially in a mechanosensory organ, 2) removal of the channel must directly eliminate mechanical response, 3) alteration of the properties of these channels must correspondingly alter the mechanical response, and 4) heterologous expression of the channel must be gated mechanically. Mechanotransduction in vocal fold tissue is particularly of interest, as vocal folds are known to be in constant oscillatory motion from airflow during voice production and homeostasis maintenance has a critical impact on their vibratory functions. Therefore, investigation of biological benefits from mechanotransduction of vocal fold tissues is a promising field of research.

Accordingly, this paper offers a narrative review of the biological effects of mechanical stimuli on soft tissues and the underlying biophysical principles of mechanotransduction. We also discuss ongoing research aimed at mimicking mechanical stimuli comparable to native vocal fold vibration to quantify the biological effects on vocal fold fibroblasts.

## Vocal fold biology

**FIGURE 1:**
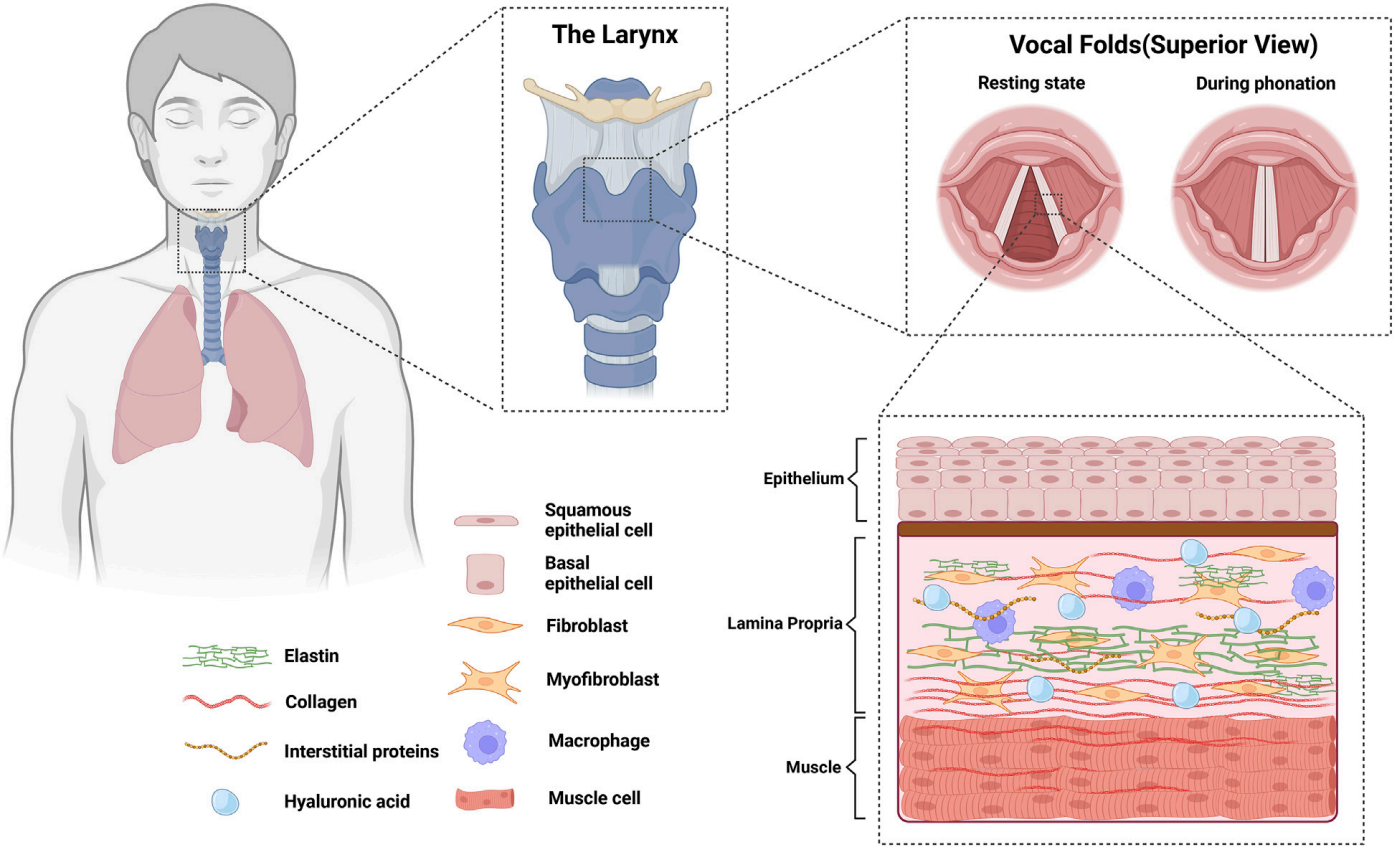
Anatomy of vocal folds and their biological constituents. Vocal folds consist of 3 biologically distinct layers (epithelium, lamina propria, and the muscle layer). Homeostasis in the lamina propria is achieved by the cells populating the area, majority of which are fibroblasts and macrophages.

The vocal fold consist of three biologically distinct layers: epithelium, lamina propria, and the muscle layer (Figure 1). The lamina propria is a connective tissue between the muscle layer and the epithelium (Hirano, 1981), populated by ECM constituents such as collagen, elastin, fibronectin, proteoglycans, and glycosaminoglycans that contribute to biological homeostasis and alter the rheological properties that affect vocal fold mucosal wave (Gray et al., 1999). In scarred vocal fold tissues, increased densities in fibronectin have been found, while decreased densities in fibromodulin and decorin were observed (Thibeault et al., 2003). Moreover, less dense and disorganized collagen fibers and decreased elastin were also found (Thibeault et al., 2002). Changes in the ECM during diseased states alter the viscoelasticity of the vocal folds (Chan and Titze, 2006). Vocal folds with higher stiffness and viscosity show less pliability, which hinders the ability to accommodate a surface mucosal wave, resulting in difficulties in achieving self-sustained oscillation. Accordingly, patients with dysphonia show disrupted mucosal wave, experience difficulties initiating phonation, and have shown increased perceived phonatory effort (Chang and Karnell, 2004; Szkiełkowska et al., 2019). Changes in ECM deposition are primarily attributed to a shift in the behavior of vocal fold fibroblasts, which are most abundantly found in the lamina propria (Catten et al., 1998; Gray, 2000). A recent transcriptomic study in an attempt to create a laryngeal cellular atlas by An et al. (2024) found similar results, showing highest abundance of fibroblasts among non-epithelial/endothelial cells of murine larynges, followed by phagocytes (primarily macrophages) and lymphocytes.

## Physical mechanisms of vocal fold oscillation

**FIGURE 2:**
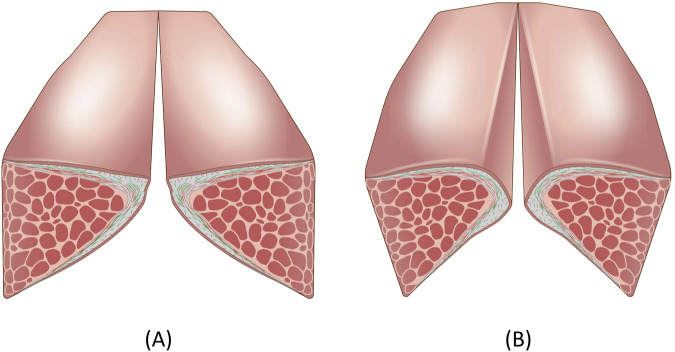
Cyclic geometrical shape changes the vocal folds undergo during oscillation. The **(A)** convergent glottis generates higher intraglottal pressure than the **(B)** divergent glottis, resulting in positive net energy transfer that sustains oscillation.

In order to understand biological benefits and drawbacks from mechanical stimulation of the vocal folds, it is important to understand how native oscillatory motion of the vocal folds occur during phonation. Vocal folds undergo self-sustained oscillation, that is, net positive energy is added into the cyclic movement of the vocal folds to compensate for the energy loss due to friction and viscous properties of vibrating tissues (Titze, 1988). Self-sustained oscillation of the vocal folds from induced airflow was first proposed by Van den Berg (1958). Van den Berg argued that increasing subglottic pressure forces the vocal folds to open, and the Bernoulli effect, which results in negative pressure in the smaller orifice, pulls the vocal folds together. However, these assumptions were refuted and refined in the 1980’s (Švec et al., 2023). The negative Bernoulli effect is a unidirectional force that is present during both glottal opening and closing phases, so it is intuitive to realize that positive net transfer from the Bernoulli effect will not occur, as the energy that is imparted to help close the vocal folds will be canceled out with the same inhibitory force when vocal folds are in the opening phase (Titze, 2019). Titze (1988) analytically showed that a positive net energy transfer is achieved through a cyclic transition from a convergent to a divergent (or from a convergent to a less convergent) shape of the glottis, resulting in varied intraglottal pressures (Figure 2). This shape change of the glottis is due to a time delay caused by the propagation of the mucosal wave velocity in the inferior-superior direction, resulting in a phase difference of displacement for the inferior and superior glottal wall (Titze, 1988; Titze and Alipour, 2006). As a result of Bernoulli’s principle of conservation of energy, intraglottal pressure is higher during a convergent glottis and lower for a divergent glottis, which exerts a pushing and pulling force on the vocal folds, facilitating oscillatory motion. Through this mechanism, aerodynamic energy is converted into kinetic movement of the vocal folds, and oscillation is sustained (Thomson et al., 2005).

Research has been extensively performed to quantify parameters that aid in self-sustained vocal fold oscillation. It was found that configuration changes of the vocal tract to achieve higher inertive properties (Titze, 2020), and configuration of the pre-phonatory glottal geometry to achieve a near rectangular glottis (Chan et al., 1997; Lucero, 1998) aid in self-sustained vocal fold oscillation. Recently, theoretical derivation, computational models, and experimental studies with canine larynges suggest that flow separation in a divergent glottis also aids in the sustained oscillation of the vocal folds by creating vortices that have a negative pressure, which aids the vocal folds during the closing phase (Farbos de Luzan et al., 2021; Sundström et al., 2022). When the above factors aid self-sustained oscillation, they reduce phonation threshold pressure (PTP), which refers to the minimum initial lung pressure required to initiate and sustain vocal fold oscillation (Titze, 1992a). Functionally, PTP is regarded as an objective measure of voice effort and voice fatigue (Titze, 1988). Higher PTP is generally unfavorable and is associated with phonotraumatic voice disorders, such as vocal fold nodules, polyps, scarring, fatigue, and during wound healing (Solomon and DiMattia, 2000; Hirano et al., 2004; Rousseau et al., 2006; Wang et al., 2010; Free et al., 2022). In contrast, lower PTP is directly linked to ease of phonation (Titze, 1992a). Titze (1988) derived an analytic expression for contributing factors to PTP for a rectangular glottis, where PTP is proportional to mucosal wave velocity, viscous damping coefficient, and pre-phonatory glottal half-width, and inversely proportional to vocal fold thickness. Of these given parameters, glottal half-width and vocal fold thickness are related to the configuration of the glottal geometry that can be adjusted with activations of the intrinsic laryngeal muscles (Vahabzadeh-Hagh et al., 2017b; Vahabzadeh-Hagh et al., 2017a; Azar and Chhetri, 2022; Pillutla et al., 2023). On the other hand, mucosal wave velocity is determined by the stiffness of the vibrating vocal fold tissue (Berke and Gerratt, 1993; Sloan et al., 1993; Titze et al., 1993), which can both be a result of biomechanical and biological changes in the vocal folds. Previous biomechanical investigations indicate that mucosal wave velocity is associated with changes in the stiffness of the vocal folds during frequency variation (Titze et al., 1993). Meanwhile, it has also been demonstrated that stiffness of the vocal folds is a dynamic parameter known to increase from biological changes resulting from phonotraumatic dysphonia as well (Jiang et al., 1998; Motie-Shirazi et al., 2023). Moreover, the viscous damping coefficient can also be determined by the biological compositions of the vocal fold mucosa, which ultimately affect its rheological properties (Chan and Titze, 1999; Gray et al., 1999; Thibeault et al., 2002). For example, it was previously demonstrated from rheological investigations from multiple studies that scarred vocal folds are generally more viscous than normal vocal folds (Rousseau et al., 2003). Therefore, biological changes in the vocal folds from phonotrauma are not only related to the decreased perceptual quality of the voice (Nuss et al., 2010; Gramuglia et al., 2014) but may directly impact vocal effort and significantly alter vocal fold vibration biomechanics due to an increase in PTP.

## Damage to vocal fold tissues from mechanical stimulation

**FIGURE 3:**
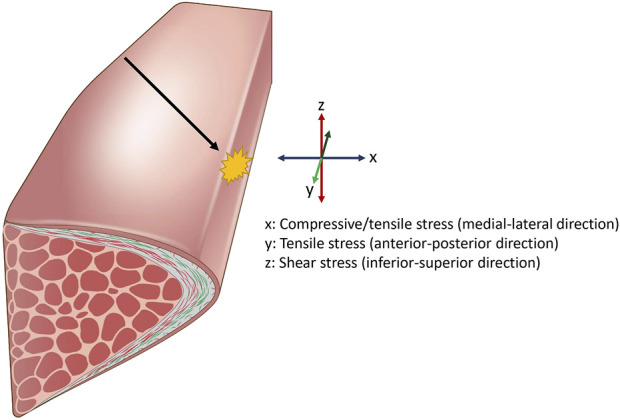
Representative stresses the vocal fold is exposed to during vocalization. Airflow displaces the vocal folds laterally, resulting in compressive and tensile stress. Airflow also displaces the vocal fold superiorly and inferiorly, resulting in shear stress on the medial surface. Pitch changes during vocalization results in elongation of the vocal fold, applying strain in the longitudinal direction. This leads to increased stress in the anterior-posterior axis. Additionally, the oscillatory motion causes the opposing vocal fold tissues to collide, generating impact stress at the site of contact. Impact stress is highest in the mid-membranous portion of the vocal fold (black arrow).

As vocal folds oscillate, they are subjected to constant stress and stimuli from air-induced force and laryngeal muscle adjustments (Titze, 1994). Some representative types of stress that vocal folds are exposed to during vibration are shown in Figure 3. Titze (1994) suggested that physiology of the vocal fold vibration has a direct causal effect to the disruption of biological homeostasis in the vocal folds that results in voice disorders. Such examples of voice disorders include the formation of benign masses of collagenous fibers such as vocal fold nodules and polyps, usually found in the mid-membranous portion of the vocal folds. It is generally believed that these masses are formed from a certain type of mechanical stress that occur during vocal fold impact, when two folds collide with each other during the oscillatory cycle. Accordingly, vocal fold impact stress have been extensively investigated with various phonatory conditions using canine larynges, human participants, and computational modeling and have shown that collision force is highest in the mid-membranous location of the vocal folds, where these benign masses are mostly seen (Jiang and Titze, 1994; Gunter, 2004; Tao and Jiang, 2007; Zhang, 2019; Motie-Shirazi et al., 2021). Jiang and Titze (1994) used canine larynges and experimentally demonstrated that pre-phonatory glottal configuration, stiffness of the vocal folds, and subglottic pressure were determining contributors to impact stress, with subglottic pressure showing a markedly high correlation. Another study further demonstrated that increased subglottic pressure results in high acceleration and deceleration of the vibrating vocal folds during larger amplitude vibration (Horácek et al., 2009), which results in a higher vocal fold collision stress due to Newton’s second law of motion (Jiang et al., 2001). More recently, computational modeling studies showed that a smaller pre-phonatory glottal angle, lower transverse stiffness of the vocal folds, higher lung pressure, and vocal tract shape with an expanded oral cavity had a causal relationship to higher impact stress (Zhang, 2019; Zhang, 2021). From a biological perspective, computational modeling and analytical investigations show that an increase in the viscosity of vibrating tissues leads to higher energy dissipation as heat during energy transfer and generally results in higher impact stress (Erath et al., 2017; Motie-Shirazi et al., 2021). Moreover, it was also suggested that edematous vocal folds cause a vicious feedback loop that results in a higher collision pressure and higher viscosity dissipation, which leads to further swelling (Deng et al., 2023). Swelling is a complex biological response of fluid buildup due to vascular leakage and disruption in the transcapillary fluid exchange, which can be caused by many factors (Gou and Pence, 2016), including excessive mechanical stimuli such as hyperfunctional voice use, which causes acute tissue inflammation and edema. It was shown that excessive vocal loading with high frequency and displacement of the vocal folds are sufficient to induce microvessel rupture from elevated intravascular pressure, resulting in an inflammatory response and leakage of fluid (Czerwonka et al., 2008). When swelling occurs, some compensatory behavioral adjustments to initiate and sustain oscillation may take place, such as a stronger glottal closure or an increase in the subglottic pressure that will again increase impact stress and cause further swelling due to high amplitude vibration. While these stresses are thought to occur during voice production, most studies that investigated stresses acting upon the vocal fold have been performed *in silico*, or from dissected animal larynges. Quantifying the exact amount of stresses during native vocal fold oscillation in humans proves to be a challenge for a number of reasons. Such examples include probes disrupting the native vocal fold vibration during measurements and insertion of such devices shifting the geometry of the vocal tract, which affects vocal fold vibratory parameters through non-linear source-filter interactions (Titze, 2008). So while computational modeling studies offer a conceptual framework of parameters related to various stresses acting on the vocal folds, precise quantities of such stresses may vary during natural vocal fold vibrations.

## Beneficial mechanical stimulation of the vocal folds *in vivo*: voice therapy

While some voice disorders are caused by large amplitude mechanical forces, there are parameters of the voice that, when changed, result in less impact stress and may prevent phonotraumatic disorders. In other words, certain vibratory stimulation of the vocal folds may be clinically and physically advantageous. Two parameters related to vocal fold oscillation and their clinical relevance have been suggested in the past: vocal efficiency and vocal economy.

Vocal efficiency is defined as the transfer ratio of aerodynamic energy to acoustic energy and is determined mathematically by subglottic pressure, glottal airflow, fundamental frequency, and maximum flow declination rate (MFDR) (Titze, 1992b). MFDR refers to the peak rate at which the glottal airflow declines during the closing phase, which is an important parameter as it is theoretically and experimentally correlated with vocal intensity (Holmberg et al., 1988; Titze and Sundberg, 1992). More recent studies indicate that vortices resulting from flow separation in a divergent glottis during self-sustained oscillation are a significant contributor to MFDR (Sundström et al., 2022). Furthermore, it has been mathematically demonstrated that narrowing the epilaryngeal airway leads to impedance matching of the source and the load, leading to maximum power transfer and increased vocal efficiency (Titze, 2021). This was also validated experimentally by Guzman et al. (2013) using a computer tomography scanner, where they found an increase in higher harmonic energy during phonation when participants narrowed their vocal tract after a semi-occluded vocal tract exercise. In this study, the investigators believed that this increase in higher harmonic energy was attributed to the narrowing of the vocal tract, which better matched the impedance of the glottal source. While vocal efficiency is informative in quantifying energy transfer, this parameter has shown little clinical value as it does not consider the cost-to-benefit ratio for vocal health (Titze, 1992b). For example, it has been shown theoretically and experimentally that a potentially hazardous “pressed voice” has a higher vocal efficiency due to low glottal airflow (Grillo and Verdolini, 2008).

On the other hand, vocal economy is defined as the ratio of the radiated acoustic power to the collision impact force of the vocal folds, which can also be mathematically expressed as the ratio of maximum flow declination rate to maximum area declination rate (Berry et al., 2001; Titze, 2006a; Titze and Laukkanen, 2007). While MFDR is indicative of acoustic output, maximum area declination rate (MADR) refers to the peak velocity rate at which the glottal area declines during the closing phase, which was proposed as an indicator that can predict the phonotraumatic impact force (Titze, 2006a). Nuances in the reliability of MADR in estimating impact stress are debated in recent studies due to findings that velocity just before impact is significantly reduced, especially at lower intensities, showing more than 95% reduction from MADR in some cases (Horáček et al., 2021; DeJonckere and Lebacq, 2022). Nevertheless, vocal economy has shown to have higher clinical relevance, as it accounts for the damage inflicted on the vocal folds during collision.

While this knowledge informs clinicians at a conceptual level of how vocal folds may be more susceptible to damage from mechanical stress, it is argued that vocal fold geometry and stiffness properties co-vary with each other, and isolated changes of these parameters would be impossible at the systemic level (Zhang, 2023). Therefore, clinicians have adapted holistic approaches to treat voice disorders and reduce impact stress, aiming to restore health in the vocal folds (Stemple, 2005). Some therapeutic approaches exist, such as Vocal Function Exercise, Lessac-Madsen Resonant Voice Therapy, Smith Accent Method, Stretch and Flow Phonation (also known as Casper-Stone Flow Phonation), that impose mechanical stimulation in certain ways that rehabilitate the vocal folds (Kotby et al., 1991; Stemple et al., 1994; Schneider and Sataloff, 2007; Verdolini-Abbott, 2008; Verdolini-Abbott et al., 2012). Within these approaches, there exists some adaptation of phonating with a semi-occlusion of the vocal tract, such as lip trills, humming, phonation into a tube submerged underwater, or singing with rounded lips that results in lower collision force of the vocal folds, thereby increasing vocal economy (Titze, 2006b; Titze and Laukkanen, 2007; Horáček et al., 2019). It is well established that such behavioral adjustment to the vocal tract geometry will shift the nature of vocal fold vibration to reduce impact stress, which serves as a scientific rationale for the aforementioned voice therapies (Titze, 2006b). Decrease in impact stress or increase in vocal economy during or after semi-occluded vocal tract exercises have been demonstrated with computational and physical models (Titze, 2006a; Titze and Laukkanen, 2007; Titze, 2008; Horáček et al., 2019; Titze, 2020), as well as experimentally (Calvache et al., 2020) using the quasi-output cost ratio (QOCR), a known parameter that indirectly quantifies vocal economy in humans participants through electroglottographic measures (Laukkanen et al., 2009).

## Biophysical explanation for mechanotransduction

**FIGURE 4:**
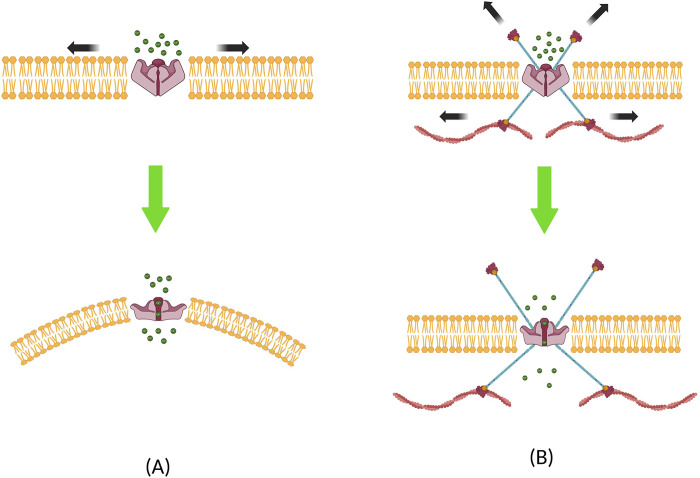
Two models that explain the biophysical principles underlying mechanosensitive channel activation. **(A)** Force-from-lipid model, where local curvature and hydrophobic mismatch causes the ion channel to open, and **(B)** force-from-filament model, where channels are activated through the influence of forces transmitted from the filaments located in the cytoskeleton or the extracellular matrix.

Voice therapies have focused on inducing mechanical stimuli in ways that are less detrimental to, or even potentially helpful to vocal fold tissues during wound repair (Verdolini-Abbott et al., 2012). It can be postulated that these stimuli are transmitted through mechanotransduction, resulting in biological changes in cellular behaviors. A brief synopsis of the underlying physical principles is presented in this section. Mechanotransduction is achieved through gating of MS channels due to external mechanical stimuli. Two models have been proposed to demonstrate the underlying biophysical principle for MS channel activation (Figure 4). The “force-from-lipids” (FFL) model shows that mechanical force applied to the lipid bilayer is the main contributor that gate the channels (Martinac et al., 1990). The FFL model can explain the gating of MS channels in the context of hydrophobic mismatch and/or the changes in the membrane curvature (Bavi et al., 2016b). When a lipid bilayer is stretched, bilayers become thinner, and MS channels embedded in the bilayers experience a hydrophobic mismatch; that is, the hydrophobic α-helices of the MS channel are now thicker than the stretched bilayer. This is followed by a conformity change in the α-helix protein to minimize the mechanical strain on the bilayer. This results in helical tilting, modulating the gated MS channels to open/closed formations (Hamill and Martinac, 2001). Local curvature changes can also induce MS channel activation. Using a finite element model, Bavi et al. (2016a) showed that local curvature radii of 50 nm are sufficient to influence the activation of MS channels. Experiments inserting amphipathic molecules into the lipid bilayer have been shown to cause such effects on TREK, TRAAK, and PIEZO1 channels (Patel et al., 1998; Syeda et al., 2016; Tsuchiya et al., 2018).

The second explanation of the underlying biophysical principle for MS channel activation can be done using the “force-from-filament” (FFF) model. This model is predicated on the assumption that the cytoskeleton and its interaction with the extracellular matrix (ECM) play a major role in mechanotransduction (Cox et al., 2019). This model was initially proposed to describe mechanotransduction in auditory and vestibular hair cells (Chalfie, 2009). In this model, filaments in the ECM and cytoskeleton are either directly tethered to MS channels or indirectly linked to the auxiliary proteins that are bound to MS channels. When tension is applied, filaments from both sides apply force on the MS channel, resulting in open/closed formations (Ranade et al., 2015; Zhang et al., 2015). Duque et al. (2022) used ultrasonic stimulation and showed that actin filaments are involved in gating the TRPA1 channel, and Prager-Khoutorsky et al. (2014) demonstrated that microtubules are involved in gating the TRPV1 channel. It should be noted that these two models explained here are not mutually exclusive, as mechanotransduction involves a dynamic interaction between both the lipid bilayers and filaments. A prime example demonstrating these mechanisms was performed on the MS TRAAK K+ channel, which was shown to be activated either from the lateral membrane tension or from the local membrane curvature (Martinac and Hamill, 2002; Aryal et al., 2017). It is now generally believed that both contribute to the activation of MS channels, and one paradigm cannot be wholly separated from the other (Cox et al., 2017, Cox et al., 2019).

## PIEZO channels and their effect on fibroblasts

Of the numerous MS channels that have been identified in the human body, the role of PIEZO channels in wound healing in fibroblasts is worth noting, as recent literature have identified PIEZO channels in the laryngeal airway and vocal fold epithelium (Lungova et al., 2020; Foote et al., 2022a; Foote et al., 2022b). PIEZO1 and PIEZO2 are non-selective cation channels, showing permeability to Na^+^, K^+,^ Ca^2+^, and Mg^2+^ ions, with a slight preference and highest affinity to CA^2+^ ions (Coste et al., 2010; Coste et al., 2012; Gnanasambandam et al., 2015). PIEZO1 is inherently mechanosensitive, suggesting that it can be activated by pure mechanical stimuli such as stretch, pressure, or shear stress (Syeda et al., 2016). PIEZO2 is closely associated with proprioception, interoception, and somatosensation in organs such as the bladder, lungs, and stomach and the sensation for arterial blood pressure (Chesler et al., 2016; Umans and Liberles, 2018; Min et al., 2019; Prescott et al., 2020; Ma et al., 2022). As PIEZO1 and PIEZO2 share similar structural properties (Saotome et al., 2018; Zhao et al., 2018; Wang et al., 2019), it was originally hypothesized that the gating mechanism for the two channels would be similar. However, PIEZO1 has been shown to modulate gating by membrane stretch alone (Cox et al., 2016), while PIEZO2 is less sensitive to membrane tension (Young et al., 2022). It was demonstrated that PIEZO2 is nonresponsive to negative pressures while being preferentially activated from positive pressures, that is, from the indentation of the membrane (Shin et al., 2019).

Research regarding PIEZO channels in fibroblasts have primarily been performed in cardiac tissues, which similar to vocal fold tissues, are also constantly exposed to mechanical stimuli (Stewart and Turner, 2021). PIEZO1 expression has been directly linked to increased fibrosis in cardiac hypertrophy by evoking Ca^2+^-mediated differentiation of fibroblasts into myofibroblasts (Beech and Kalli, 2019; Zhang et al., 2021). Blythe et al. (2019) found that through a PIEZO1 agonist-mediated activation in murine cardiac fibroblast, a 2–4 fold increase in IL-6 gene expression and a 3–4 fold protein increase in p38 MAPK were detected, indicating that PIEZO channels may play a contributing role in cardiac remodeling that may result in cardiac fibrosis (Ma et al., 2012). From the above studies, it is shown that cardiac fibroblasts play an important role in mediating inflammation and fibrosis. Generalizing the behavior of cardiac fibroblasts when exposed to mechanical stimuli to human vocal fold fibroblasts is premature, given the demonstrated heterogeneity in gene expression across fibroblasts from different tissue types (Foote et al., 2019). A recent study by Zheng et al. (2024) investigated the role of PIEZO1 in lung fibroblasts, which is more similar in transcriptome profiles to vocal fold fibroblasts. In this study, the investigators exposed human lung fibroblast cells to static tensile stress, and found protein upregulation in α-SMA, Col1a1, and fibronectin in fibroblasts with functioning PIEZO1 channels, compared to fibroblasts with PIEZO1 inhibited or knocked out. This suggests that attenuation of PIEZO1 expression may be a potential therapeutic target in curing fibrosis. Given that PIEZO channels mediate cellular responses in such ways that may potentially be critical in vocal fold physiology, further investigation of these mechanosensitive channels in vocal fold cells could provide clinically relevant insights.

To our knowledge, only three studies have investigated PIEZO channels in the vocal folds. Studies localizing the PIEZO channels in the vocal folds showed that PIEZO1 is present in epithelial cells of the mid-membranous vocal folds in mice, while PIEZO2 were present in the sub- and supra-glottic regions (Foote et al., 2022a; Foote et al., 2022b). While much remains to be uncovered, recent studies indicate that the life cycle of vocal fold epithelial cells is highly dependent on PIEZO1, similar to findings in renal epithelial tissues (Eisenhoffer et al., 2012; Gudipaty et al., 2017). Lungova et al. (2020) found that activation of PIEZO1 in epithelial cells of the vocal folds may contribute to downstream signaling that results in vocal fold epithelial stratification and changes in ECM stiffness. Foote et al. (2022a) also found that PIEZO1 contributes to epithelial remodeling after a chemical-induced vocal fold injury. Meanwhile, PIEZO1 contributions in response to mechanical stress are unknown and remain an objective for future investigations.

Moreover, PIEZO channels have shown to be dynamically expressed in fibroblasts depending on the substrate property on which cells were seeded. Braidotti et al. (2024) found that in a softer polydimethylsiloxane substrate, cardiac fibroblasts express lower levels of the PIEZO1 gene, ultimately inhibiting differentiation into myofibroblasts. Similar results were also shown by He et al. (2024), where human dermal fibroblasts seeded in substrates with higher mechanical stiffness resulted in higher expression levels of PIEZO1, which ultimately led to increased fibrotic response. These results are especially of interest because, unlike other organs, the vocal folds constantly undergo dynamic changes in stiffness and geometry during vocalization upon activation of the thyroarytenoid (TA), cricothyroid (CT), and lateral cricothyroid (LCA) muscles (Titze, 2014; Vahabzadeh-Hagh et al., 2017b). In practice, changes in the fundamental frequency and the sound pressure level were shown to have a direct cause-effect relationship with vocal fold stiffness (Zhang, 2017). Furthermore, vibratory patterns observed during modal and falsetto phonation is thought to be dependent on the differential stiffness properties along the superior-inferior axis of the vocal folds, mediated by the TA and LCA muscles (Titze, 2014). Moreover, stiffness variations along the superior-inferior axis of the vocal folds have been observed, with inferior portions exhibiting higher stiffness in both canine and human vocal folds (Chhetri et al., 2011; Chhetri and Rafizadeh, 2014). This heterogeneous stiffness disparity increases under tensile strain, which would occur through the activation of the CT muscle *in vivo* (Oren et al., 2014). This implicates that depending on vocalization habits, localization and expression of PIEZO1 in fibroblasts for individuals may differ substantially. While PIEZO expressions in vocal fold fibroblasts have not yet been directly investigated, future work regarding this topic shows promising implications that advance our understanding of how voice disorders develop in humans.

## Anti-inflammatory effects of mechanotherapy on soft tissues and fibroblasts

Advances in knowledge of mechanotransduction and mechanobiology facilitated the development of new therapies that utilize mechanical stimulation to manipulate cell behavior, ultimately leading to tissue or organ repair. It has been demonstrated at the cellular level that disrupting the mechanical equilibrium by inducing force could promote tissue regeneration (Lo et al., 2000; Engler et al., 2006). Extracorporeal shockwave therapy (ESWT), a form of mechanotherapy, induces a transient burst of positive pressure, rapidly followed by a variable lower pressure (Ogden et al., 2001). These acoustic waves are detected by (MS) channels in cells, which alter cellular behavior, leading to various biological benefits. These benefits include reduced wound size (Schaden et al., 2007), accelerated reepithelialization (Ottomann et al., 2012), enhanced angiogenesis (Huang et al., 2017; Sundaram et al., 2018), and increased fibroblast proliferation and collagen synthesis (Yang et al., 2011). Cui et al. (2018) found dermal fibroblasts harvested from human scar tissue stimulated with ESWT showed lower gene expression and protein production levels of TGF-β1, α-SMA, fibronectin, and Col1a1 compared to controls after 24 h, implicating anti-fibrotic effects. Meanwhile, Berta et al. (2009) reported that normal human dermal fibroblasts showed an increase in TGF-β1 gene expression 6 and 9 days after ESWT, an increase in collagen I gene expression 12 days after ESWT, and an increase in collagen III gene expression 9 and 12 days after ESWT with a significant decrease on day three compared to the control group. Discrepancies in the biological responses indicate that cells respond selectively to different parameters of mechanical stimuli (Romeo et al., 2014). ESWT dose-dependent cell behaviors were quantified, and it was shown that ESWT can manifest both pro-inflammatory and anti-inflammatory effects depending on the energy of the shockwave and the number of pulses of shocks that were applied (Zhang et al., 2014). ESWT has been shown to exhibit changes in cellular response of macrophages as well. Polarization of macrophages to the anti-inflammatory phenotype M2 macrophages were detected, with higher protein expression of anti-inflammatory IL-10 secretion after ESWT (Abe et al., 2014; Tepeköylü et al., 2015). It was also shown that ESWT stimulation for 4 h resulted in a significant downregulation in gene and protein expression of inflammatory markers such as IL-1β, CCL5, CXCL9, and CXCL10 from M1 macrophages (Sukubo et al., 2015).

The effects of ultrasonic irradiation have also been studied extensively on soft tissues. Using low-intensity pulsed ultrasound (LIPUS) has been found to increase protein expression of MS channels, such as PIEZO1, Cav1.2, NCX1, TRPV1, and TRPM7 (Zuo et al., 2018; Yao et al., 2022; Zheng F. et al., 2024), promote cell proliferation (Kobayashi et al., 2009; Puts et al., 2018; Tan et al., 2021), ameliorate fibrosis (Aibara et al., 2020; Kun et al., 2021; Zhao et al., 2021; Zhou et al., 2023), and promote ECM remodeling (Zhang et al., 2016). In fibroblast cells, Zhou et al. (2004) showed that 6 and 11 min of LIPUS exposures in human dermal fibroblasts resulted in significant fibroblast proliferation after 24 h. Franco de Oliveira et al. (2011) found similar results of higher proliferation compared to the control group after 24, 48, 72, and 96 h in murine lung fibroblasts. Moreover, Perrucini et al. (2021) found that FGF7 and VEGF genes related to angiogenesis were upregulated and found increased Col1a1 gene expression and decreased IL-6 inflammatory gene expression after 48 h of LIPUS stimulation. Weng et al. (2022) found anti-inflammatory and anti-fibrotic effects of LIPUS on neonatal rat cardiac fibroblasts, showing downregulation of NLRP3-related inflammatory response and downregulation of NOX4, implying decreased oxidative stress. Macrophages have also shown behavioral changes in response to LIPUS. Xu et al. (2023) found that after 6 weeks of LIPUS exposure, approximately 3.5-fold and 5.5-fold upregulation in anti-inflammatory IL-10 and CD163 gene expression were measured, respectively. In this study, the investigators also measured the phenotype changes of macrophages by quantifying the ratio of M1 to M2 macrophages and found higher amounts of M2 macrophages after LIPUS exposure. Similar to the polarization changes in macrophages from ESWT exposure, polarization to M2 macrophages from LIPUS stimulation was also reported in other studies (da Silva Junior et al., 2017; Zhang et al., 2019). Suppression of inflammatory effects was also seen in a study by Feltham et al. (2021), where they found 5.2-fold downregulation in protein expressions of IL-1β and suppression of CD68^+^ macrophage infiltration after LIPUS stimulation in rats with intra-articular fracture at the tibial plateau. Finally, a study by Iacoponi et al. (2023) showed downregulation of gene and protein expression of TNF-α, IL-1β, and IL-8 that was shown to vary from dosage and frequency of LIPUS. In this study, they also used mechanosensitive channel blockers to suggest that protein expression of TNF-α, IL-1β, IL-4, IL-6, IL-8, IL-10, and IL-12p70 may be regulated by PIEZO and TRP channels.

When investigating mechanotherapy at the systemic level, massage therapy has been associated with anti-inflammatory and anti-fibrotic benefits. When investigating massage with inflammation, Crane et al. (2012) found that a 10-minute massage therapy immediately after exercise showed the immediate effect of decreased expression in NFκB protein content in a diet-controlled study with 11 human participants. Correspondingly, the protein content of pro-inflammatory cytokine IL-6 was also downregulated 2.5 h later, indicating that mechanotransduction occurred rapidly in response to stretch, leading to anti-inflammatory effects. White et al. (2020) found similar anti-inflammatory effects of massage therapy in 9 athletes, where they reported quicker downregulation of TNF-α, IL-6, IL-8, and MCP-1 after loading compared to the control group. Rats exposed to massage therapy have shown decreased protein levels of pro-inflammatory markers such as IL-1β, IL-18, MCP-1, and TNF-α, increased protein levels of anti-inflammatory marker IL-10, and inhibition of fibrotic response (Barbe et al., 2021a; Barbe et al., 2021b). At a practical level, meta-analyses of soft tissue mobilization after mechanical loading have been shown to relieve pain and improve range of motion and flexibility (Cheatham et al., 2016; Davis et al., 2020). However, high variation and lack of rigorous controls in methodology and investigated parameters used across studies indicate that comparisons across studies are unreliable (Lima et al., 2020), arguably very similar to voice therapy studies.

As mentioned above, mechanotherapy encompasses a wide range of therapeutic approaches, each inducing diverse biological changes that are beyond the scope of this review. For a comprehensive examination of the recent advances in specific mechanotherapies, readers are encouraged to refer to the following review articles (Frairia and Berta, 2011; Jiang et al., 2019; Lima et al., 2020; Xu et al., 2021; Qin et al., 2022). However, it can be seen from various studies that biological outcomes of mechanical stimuli are highly dependent on cell type, treatment method, and duration, as well as other technical discrepancies among different experimental designs (Eagan et al., 2007; Franco de Oliveira et al., 2011; Zhang et al., 2014; Tan et al., 2021; Weng et al., 2022; Iacoponi et al., 2023). It is apparent that cells are highly sensitive to various mechanical stimuli, and minute changes in parameters, such as frequency, amplitude, or applied duration, can result in heterogeneous cell behaviors. For example, in a study to find the optimum parameters for ESWT on endothelial progenitor cells, it was shown that using the energy of 0.04 mJ/mm^2^ per impulse, 60 more impulses of shock waves from 140 impulses to 200 impulses resulted in an approximate 30% downregulation of IL-6 gene expression, while at a fixed rate of 140 impulses, increasing the energy from 0.07 to 0.1 mJ/mm^2^ resulted in an approximate 40% downregulation of IL-6 gene expression (Zhang et al., 2014). Additionally, dermal fibroblasts subjected to cyclic short-duration stretches designed to replicate the effects of repetitive motion injury exhibit an inflammatory response. In contrast, strain models with changes in some parameters that simulate the mechanical effects of massage therapy have been shown to reverse this response (Meltzer and Standley, 2007; Meltzer et al., 2010; Anloague et al., 2020). These findings indicate that precise modulation of mechanical stimulation parameters can elicit markedly different cellular outcomes.

## Mechanical stimulation of fibroblasts *in vitro* using vocal fold mimetic bioreactors

**FIGURE 5:**
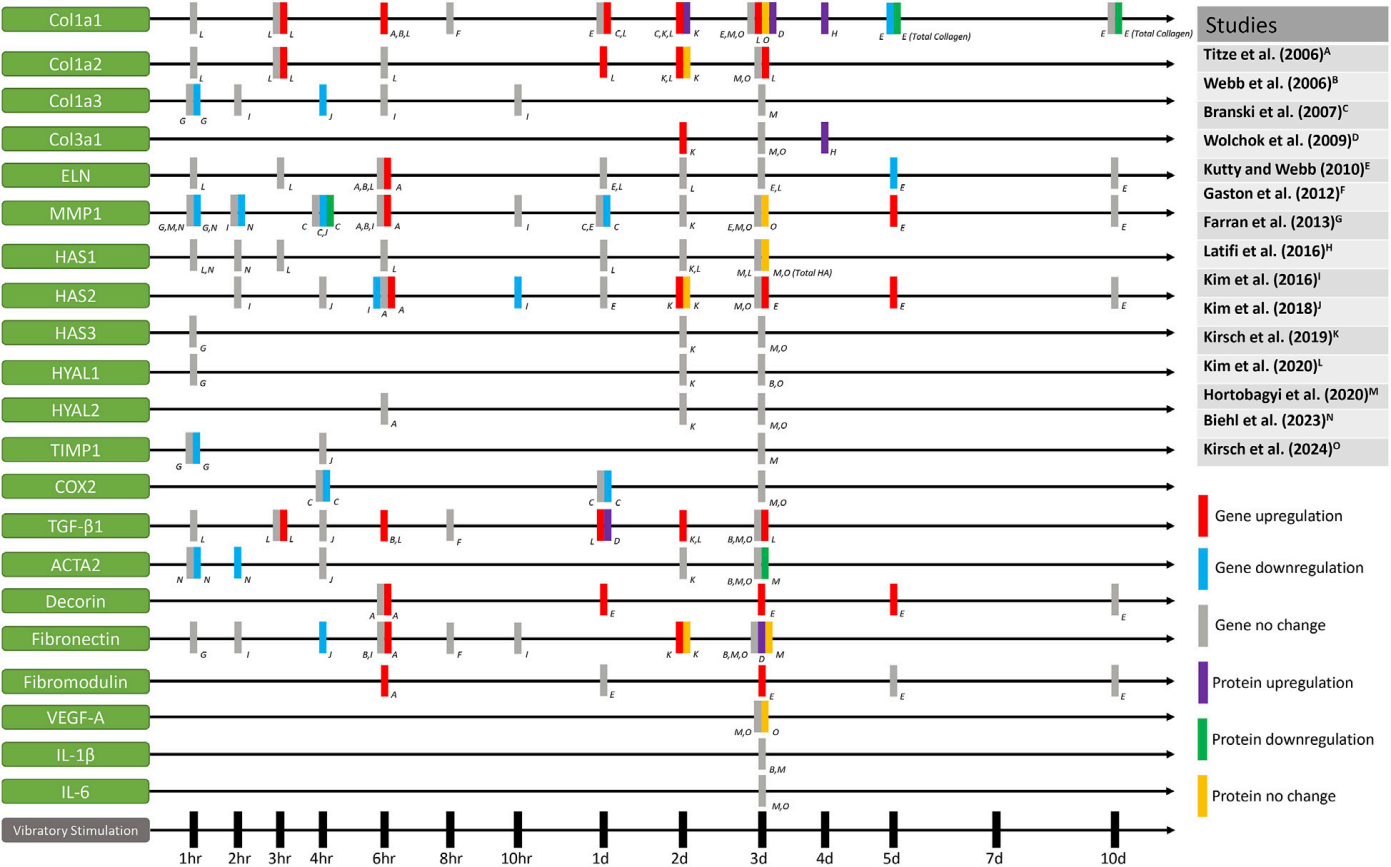
Gene and protein expression of fibroblasts that have been mechanically stimulated by vocal fold mimetic bioreactors.

It can be postulated that fibroblasts in the vocal fold lamina propria will exhibit distinct behaviors in response to various mechanical stress. Due to difficulties controlling unknown variables among human participants, researchers have turned to using bioreactors to impose mechanical force on vocal fold fibroblasts and examine the differentiated cell behaviors. Table 1 shows specific details of the studies exposing fibroblasts to vocal fold mimetic movement, and Figure 5 shows the corresponding outcomes. For a recent comprehensive review solely focused on vocal fold mimetic bioreactors, readers are referred to Gracioso Martins et al. (2022).

**TABLE 1:** Summary of study designs implemented in mechanical stimulation of fibroblasts using vocal fold mimetic bioreactors.

	Fibroblast source	Substrate type	Stimulation frequency	Strain	Stimulation type	Stimulation pattern and duration
Titze et al. (2004)	Human laryngeal fibroblasts	Tecoflex substrate coated with fibronectin	100 Hz	20% tensile strain	Continuous vibration	6 h
Webb et al. (2006)	Human tracheal fibroblasts	Tecoflex substrates coated with bovine plasma fibronectin	0.25 Hz	10% tensile strain	Cyclic strain	6 h
Branski et al. (2007)	Rabbit vocal fold fibroblasts	Collagen type I-coated Bioflex II plates	0.005/0.05/0.5 Hz	3/6/9/18% tensile strain	Cyclic strain	4/24/48 h of cyclic strain
Wolchok et al. (2009)	Human laryngeal fibroblasts	Polydimethylsiloxane and polyurethane scaffolds coated with fibronectin	100 Hz	No strain	Pulsed vibration	1.5 s on/30 s off for 6 h, rest for 18 h per day for total of 3 days
Kutty and Webb (2010)	Human dermal fibroblasts	Methacrylated hyaluronic acid hydrogels crosslinked to Tecoflex films	100 Hz	No strain	Pulsed vibration	2 s on/2 s off for 4 h per day for total of 10 days
Gaston et al. (2012)	Immortalized human vocal fold fibroblasts	Tecoflex substrate coated with human fibronectin	200 Hz	20% tensile strain	Continuous vibration/strain	8 h of continuous vibration
Farran et al. (2013)	Primary human neonatal foreskin fibroblasts	Collagen I – coated silicone membranes	60/110/300 Hz	No strain	Continuous vibration	1 h of continuous vibration
Latifi et al. (2016)	Human vocal fold fibroblasts	Thiol-modified heparin-bonded hyaluronic acid and thiol-modified gelatin hydrogel cross-linked with polyethylene glycol acrylate	Approx. 100 Hz	No strain	Pulsed vibration	1 h on/15 min off/1 h on for a total of 4 days
Kim et al. (2016)	Human vocal fold fibroblasts	Bioflex flexible culture plate coated with type I collagen	205 Hz	No strain	Continuous vibration	2/6/10 h of continuous vibration
Kim et al. (2018)	Human vocal fold fibroblasts	Bioflex flexible culture plate coated with type I collagen	205 Hz	No strain	Pulsed vibration	2 s on/2 s off for 4 h, with 1 h rest before analyses
Kirsch et al. (2019)	Immortalized human vocal fold fibroblasts	Bioflex flexible culture plate coated with pronectin	50–250 Hz sweep	No strain	Pulsed vibration	1 min on/1 min off for 16 h for total of 2 days
Hortobagyi et al. (2020)	Immortalized human vocal fold fibroblasts	Bioflex flexible culture plate coated with pronectin	50–250 Hz sweep	No strain	Continuous vibration	4 h of continuous vibration per day for total of 3 days (72 h) and 1 h of rest before analyses
Kim et al. (2021)	Primary vocal fold fibroblasts	Corning T75 flask with a hydrophilic, negatively charged polystyrene surface, modified by corona discharge treatment	50/80/100/130 Hz	No strain	Continuous vibration	0/1/3/6/24/48/72 h of continuous vibration
Biehl et al. (2023)	human vocal fold fibroblasts	Tissue culture plastic treated flasks coated with Tegaderm dressing	100 Hz	No strain	Continuous vibration	1 and 2 h of continuous vibration
Kirsch et al. (2024)	Immortalized human vocal fold fibroblasts	Bioflex flexible culture plate coated with pronectin	100–135 Hz sweep/200–250 Hz sweep	No strain	Continuous vibration	30 s on/90 s off for 16 h per day for total of 3 days

Similar to studies that have investigated other cells stimulated with mechanical force, the cellular behaviors of vocal fold fibroblast in response to mechanical stimulation are heterogeneous and dependent on the specific parameters of the vibratory forces. Nevertheless, it is shown in Figure 5 that proteins or genes related to collagen synthesis and ECM remodeling are generally upregulated when exposed to mechanical stimulation. Higher levels of collagen I and collagen III gene expression typically show up to 3 days from an initial dosage of mechanical stimulation. This may indicate that while nuanced behaviors of vocal fold fibroblasts are stimuli-dependent, ECM turnover seems to be accelerated compared to static vocal fold fibroblasts to remodel the ECM faster when exposed to mechanical stimulation.

Moreover, the investigation of pro/anti-inflammatory effects of mechanical stimulation is also worth discussing from Figure 5. Of the reviewed literature, Hortobagyi et al. (2020) and Branski et al. (2007) treated the fibroblasts with pro-inflammatory cytokines to investigate the effect of mechanical stimulation on vocal fold fibroblasts that mimic the state of acute inflammation. Hortobagyi et al. (2020) reported that human vocal fold fibroblasts treated with pro-inflammatory cytokines resulted in downregulation of IL-11 gene expression level after mechanical stimulation. Their results demonstrated that mechanical stimulation may have anti-inflammatory effects and downregulation in fibrotic responses of vocal fold fibroblasts, as IL-11 is a critical regulator in fibrosis (Ng et al., 2020; O’Reilly, 2023). Meanwhile, Branski et al. (2007) showed that cyclic strain of the rabbit vocal fold fibroblasts resulted in inhibition of COX-2, iNOS, and MMP-1 induced by IL-1β, implicating anti-inflammatory effects. In the context of inflammation suppression, Wolchok et al. (2009) found a significant decrease in the pro-inflammatory MCP-1 protein expression in normal human laryngeal fibroblasts. These three results demonstrate that anti-inflammatory effects are elicited through various pathways, the specifics of which may also depend on the parameters of the mechanical stimuli.

Higher gene expression of fibromodulin and decorin were also shown when induced with mechanical stimulation at various time points (Titze et al., 2004; Kutty and Webb, 2010). Although there were only two studies that investigated the levels of fibromodulin and decorin, this may implicate that stimuli-dependent ECM collagen turnover may take place without the formation of scar tissues, as scarred vocal folds are known to show decreased expression of fibromodulin and decorin (Thibeault et al., 2003). Fibromodulin is known to modulate the TGF-β1 activation pathway by reducing scar formation and decreasing expression of the ACTA2 gene (Zheng et al., 2017), a known biomarker for myofibroblast differentiation. Meanwhile, decorin is also known to inhibit TGF-β1 production, a cytokine responsible for scar formation (Zhang et al., 2007). The effect of reduction in fibrous molecules on vocal fold vibration will be discussed further below.

Comparing the results of the above studies at face value prove to be a challenge as experimental designs varied heavily among studies. Notable differences include frequency, amplitude, duration of stimulations, and rest intervals. Most studies did not apply continuous stimulation to cells; instead, they included periods of rest, during which cells remained static within the bioreactors. Furthermore, some studies used the same bioreactor with varying vibratory parameters or pre-stimulatory cytokine treatment, leading to differences in gene expression even in same time points. Accordingly, it is worth noting some inconsistencies and conflicting results in gene expression for same time points, as seen in Figure 5. For example, researchers found significant decrease and no changes in MMP1 gene expression after 1 h, 2 h, and 1 day of stimulation. Conflicting results in MMP1 gene and protein expression are also reported at the 6 h time point and 3 days after stimulation. Collagen, elastin, HAS2, TIMP1, COX2, TGF-β1, ACTA2, decorin, and fibronectin gene expression levels vary when analyzed at same time points, showing significant increase, decrease, and/or no changes. Discrepancies in the results may be attributed to dose-dependent cellular responses, as well as the various sources of fibroblasts extracted from other animals or other parts of the body. Moreover, the mechanical properties of the used substrates may have also affected cell behavior, as substrate stiffness is known to change collagen synthesis and pro-inflammatory behavior in fibroblasts (Tiskratok et al., 2023; Verma et al., 2023; Felisbino et al., 2024; He et al., 2024). Most importantly, stimulation frequency, type, and duration were different across all studies, and even within some of the reviewed literature, fibroblasts have shown different behaviors depending on the frequency, intensity, or duration of the mechanical stimuli. Considering the parameter-dependent heterogeneous cell behaviors that were also shown for macrophages in other mechanotherapy studies mentioned above (Davis et al., 2009; Sukubo et al., 2015; Zhang et al., 2017; Zhang et al., 2019; Feltham et al., 2021; Gouda et al., 2023; Iacoponi et al., 2023; Xu et al., 2023), it is postulated that fibroblasts and macrophages in the vocal fold lamina propria will also likely exhibit distinct behaviors in response to varying forms of mechanical stress.

Furthermore, the biological outcomes from above bioreactor studies may not translate to the systemic level, as it was also demonstrated that fibroblast cells alone may not represent the complexity of their behavior *in vivo*. Specifically, the vocal fold epithelial cells will also be stimulated by mechanical stress, which may affect the behaviors of vocal fold fibroblasts. It was shown that in other parts of the human body, mechanical stress such as hydrostatic pressure, compressive force, and shear stress affect epithelial cells to express TGF-β1, TGF-β2, MMP-9, and TIMP-1 that mediate fibroblast behavior that may ultimately affect fibrotic depositions and matrix remodeling in the ECM (Swartz et al., 2001; Tschumperlin et al., 2003; Caracena et al., 2022). As MS channels TRPV3/4 and PIEZO1/2 have been identified in the vocal fold epithelia (Foote et al., 2022a), it is not unreasonable to assume that mechanical stimuli will also affect vocal fold epithelial cells that may cause changes in the downstream pathways for vocal fold fibroblasts.

## Changes in vocal fold biology and their impact on vocal fold vibration

Overexpression of fibrotic depositions is a well-documented phenomenon that can be attributed to the activation of the TGF-β1 that ultimately leads to fibrosis (Meng et al., 2016; Hu et al., 2018). Upon exposure to TGF-β1, fibroblasts differentiate into myofibroblasts, characterized by secretion of α-SMA, which is considered a reliable marker for myofibroblast differentiation (Darby et al., 1990; Serini and Gabbiani, 1999). It has been shown that vocal fold fibroblasts treated with TGF-β1 share a similar phenotype with myofibroblasts that have been isolated from scarred vocal folds (Branco et al., 2016), and correspondingly α-SMA has also been found in vocal fold scar tissues as well (Jetté et al., 2013). Myofibroblasts undergo higher collagen production than regular fibroblasts (Lijnen and Petrov, 2002), and excessive collagen deposition has been found in scarred vocal folds (Hirano et al., 2009), which changes the viscoelastic properties of the vocal folds to become more stiff and viscous (Rousseau et al., 2004). Physically, an increase in dynamic viscosity results in higher energy loss (typically dissipated as heat energy) in the viscoelastic vocal fold tissue, which would require more expenditure of energy to sustain vocal fold oscillation (Finkelhor et al., 1988; Chan and Titze, 1999; Gray et al., 1999), ultimately increasing the PTP. Moreover, increase in the stiffness of the vocal folds also changes vocal fold oscillation properties (Alipour et al., 2000; Berry, 2001), and subsequently raises the PTP as well (Chan and Titze, 2006). As TGF-β1 levels are indicative of potential pro-fibrotic changes in the vocal folds, which changes its rheological properties, this may be the reason why investigation of TGF-β1 and α-SMA encoding gene ACTA2 levels has been of interest in past literature. Hortobagyi et al. (2020) reported that human vocal fold fibroblasts treated with inflammatory cytokines prior to mechanical stimulation did not significantly alter TGF-β1 gene expression levels but downregulated protein levels of α-SMA of more than 50% after mechanical stimulation, while showing no changes in both TGF-β1 and ACTA2 gene expression levels for untreated human vocal fold fibroblasts after mechanical stimulation. Similar results were seen by Kirsch et al. (2024), where they found no significant differences in both TGF-β1 and ACTA2 gene expression levels in untreated human vocal fold fibroblasts after vibratory stimulation for 3 days. Gaston et al. (2012) also found no significant changes in TGF-β1 gene expression levels in normal human vocal fold fibroblasts after 1 day of vibratory stimulation. On the other hand, Biehl et al. (2023) found a downregulation in ACTA2 gene expression 1 and 2 h after stimulation. Interestingly, Webb et al. (2006) and Kirsch et al. (2019) reported a significant increase in TGF-β1 gene expression after 6 h and 2 days of vibratory stimulation, respectively. Furthermore, Wolchok et al. (2009) reported an increase in TGF-β1 protein expression 24 h after vibration was applied.

Given that TGF-β1 is a stimulant to fibroblasts for signaling collagen production (Lijnen and Petrov, 2002; Petrov et al., 2002), it seems reasonable that TGF-β1 may be upregulated along with collagen synthesis. However, physiologically relevant measures of whether this upregulation of TGF-β1 reported in above studies will invoke fibrosis at a tissue level remain unknown. Rheological investigations must be considered to investigate whether biological changes such as fibrosis affect the systemic physiology of vocal fold vibration. To date, only one study by Kutty and Webb (2010) has investigated the rheological properties of the cell-seeded substrate after mechanical stimulation. Although they did not examine changes in elastic properties (Young’s or elastic shear modulus), their study found that dynamic viscosity decreased after mechanical stimulation, particularly when a higher number of cells were initially seeded into the substrate (4 × 10^6^ cells compared to 2 × 10^6^ cells and acellular condition). This study’s results indicated that vocal fold fibroblasts behavior significantly affected the substrate property, leading to a macrostructural property reduced dynamic viscosity. To summarize, results from prior studies suggest that mechanical stimulation results in reduced inflammatory effects from initial pro-inflammatory and pro-fibrotic cytokine treatments. Additionally, mechanical stimulation causes changes in the fibroblast behaviors that decrease the macrostructural dynamic viscosity of the substrate on which fibroblasts are seeded, which can be physiologically advantageous in the case of vocal fold vibration.

Furthermore, hyaluronic acid (HA) expression changes may also impact vocal fold function. HA, encoded by HAS genes, is a glycosaminoglycan with a unique ability to attract and bind to water molecules up to 1000-fold its molecular weight. Therefore, HA has been associated with improved hydration, demonstrating higher levels of water content from chronometer measurement in the dermis after HA injections (Seok et al., 2016; Choi et al., 2020). Due to this nature of attracting water molecules, HA is known to absorb mechanical forces and change the viscoelastic property of the vocal folds. The role of fluid and hydration in vocal fold tissues regarding impact stress and implications in pathologies have been extensively investigated. With the knowledge that the lamina propria is structurally similar to other porous, permeable tissues in the body, Zhang et al. (2008) introduced a biphasic model consisting of solid and liquid components of the vocal folds to investigate the dynamics of solid-fluid interactions during vocal fold vibration. In that study, they found that interstitial fluid provides stress support comparable to the solid components of the vocal folds during stress relaxation, implying the importance of hydrated vocal folds. Experimental evidence has demonstrated that systemic dehydration of the vocal folds alters their viscoelastic properties, leading to higher viscosity and stiffness (Chan and Tayama, 2002; Yang et al., 2017). Chan and Tayama (2002) studied canine larynges and induced systemic dehydration through osmosis by immersing vocal fold tissue samples in 25% sucrose solution for 30 min, and observed increases in both elastic shear modulus and dynamic viscosity. Similarly, Yang et al. (2017) used canine larynges, but instead dehydrated vocal fold tissues using a vacuum dryer. By normalizing dehydration levels to the maximum point at which the net weight of vocal fold samples no longer changed, they found that vocal folds became stiffer as dehydration increased to 40%, 60% and 80%, respectively. From these studies, it appears that changes in viscoelastic properties from dehydration alone may have minimal impact on vocal fold physiology, as such dehydration levels are highly unlikely to occur in living human tissues. Wu and Zhang (2024) used a computation model to demonstrate that within a physiologically relevant range, dehydration-induced stiffness showed almost no impact on vocal fold physiology such as changes in closed quotient, fundamental frequency, and sound pressure level. Instead, it has been hypothesized systemic dehydration may significantly impact muscle physiology such as changes in endurance and increase in fatigue which could lead to compensatory behaviors that affect voice production (Wu and Zhang, 2017). While dehydration itself may not substantially affect vocal fold physiology, fluid presence and dynamics within vocal folds may be critical biomechanical factors that contributes to vocal fold pathology (Erath et al., 2017). Studies using poroelastic models revealed that fluid migrates to the mid-superior portion of the vocal fold during sustained phonation, which results in localized edema at the mid portion of the vocal folds (Tao et al., 2010; Scholp et al., 2020). It was hypothesized that fluid accumulation in this area increases the pore pressure sufficiently to trigger an inflammatory response (Kvit et al., 2015). However, it is also thought that this fluid migration could potentially absorb the impact force and protect solid components of the vocal folds from damage over a single oscillation cycle. Scholp et al. (2020) and Tao et al. (2013) demonstrated that during a single-cycle oscillation, fluid is accelerated rapidly toward the midline of the vocal folds just before the collision and quickly dissipates away from the midline just after the collision. Therefore, a portion of the impact force energy has to be distributed to accelerate the fluids, thereby decreasing the impact force. As HA is crucial in attracting water molecules, it is presumed that the presence of HA will contribute to the fluid dynamics of vocal fold vibration. It was shown that removing HA in the vocal folds through enzymatic degradation increased dynamic viscosity (Gray et al., 1999; Chan et al., 2001), while HA-derived biomaterials injected into wounded rabbit vocal folds resulted in a decrease in viscous modulus (Duflo et al., 2006; Thibeault et al., 2011). Results from these studies emphasize the importance of fluid dynamics in vocal fold contact stress, with HA potentially acting as a key mediator. Although the results from vocal fold mimetic bioreactors show conflicting results for HAS gene expression, consistent results of no expression changes in HYAL genes responsible for HA degradation and evidence of upregulated HAS genes may suggest biological advantages of mechanical stimulation of vocal fold tissues. In the reviewed literature, there are some indications that subjecting vocal fold fibroblasts change gene expression levels of HAS2. Like many other investigated genes or proteins, some studies failed to find any differences, which further supports that expression of HAS gene may be determined by not only the presence or absence of mechanical stimuli but also how they are induced.

## Conclusion

Vocal folds are highly mobilized organs in the human body, utilized daily and subjected to numerous mechanical stimuli. Mechanical stimuli are transmitted through MS channels that are expressed on the surface of cells, which bring about changes in gene expressions, leading to a variety of heterogeneous cellular responses. It is shown that various mechanical stimuli to vocal fold fibroblasts resulted in heterogeneous cellular behaviors, as shown in a vast body of literature for cells in other parts of the human body. With growing evidence supporting the beneficial effects of vocal fold mobilization, linking the physiology of voice production with cellular biological responses has become a critical focus for voice researchers. Therapeutic approaches beyond traditional voice therapy—those that induce vibration at known frequencies and amplitudes—may offer valuable insights into benefits of mechanotransduction of vocal folds. Given that cellular responses vary widely from nuanced parameter changes during mechanical stimulation, a thorough investigation into the mechanotransduction of vocal fold fibroblasts could be critical for understanding wound healing and the maintenance of vocal fold homeostasis.
